# The Retrospective Molecular Analysis of Large Or Giant Congenital Melanocytic Nevi in a Group of Polish Children

**DOI:** 10.34763/jmotherandchild.20212501.d-21-00007

**Published:** 2021-10-11

**Authors:** Katarzyna Wertheim-Tysarowska, Orest Szczygielski, Katarzyna Seliga, Andrzej Tysarowski, Jerzy Bal, Elżbieta Michalak, Agnieszka Magdalena Rygiel, Ewa Sawicka

**Affiliations:** 1 Institute of Mother and Child, Medical Genetics Department, Kasprzaka 17a, PL 01-211, Warsaw, Poland; 2 Clinic of Surgery of Children and Adolescents, Kasprzaka 17a, PL 01-211, Warsaw, Poland; 3 Maria Sklodowska-Curie Memorial Cancer Center and Institute of Oncology, Translational and Molecular Oncology Department, W. K. Roentgena 5, PL 02-781, Warsaw Poland; 4 Institute of Mother and Child, Department of Pathology, Kasprzaka 17a, PL 01-211, Warsaw, Poland

**Keywords:** Congenital melanocytic nevi, CMN, GCMN, NRAS

## Abstract

**Background:**

Large and giant congenital melanocytic nevi (CMN), benign naevomelanocytic proliferations derived from neural crests, with a projected adult size (PAS) ≥ 20 cm, are connected to a high risk of melanoma and neurocutaneous melanosis. Among several factors, genetic alterations seem to be involved in tumorigenesis. The aim of the present study was to analyse the mutation status of *NRAS* and *BRAF* genes in resection specimens from large or giant CMN in a group of Polish patients.

**Material and methods:**

The formalin-fixed, paraffin-embedded resection specimens from 18 patients, fixed in the years of 2006 to 2017, were included in the study. The regions containing the highest load of melanocytes were macrodissected prior to DNA isolation. The *NRAS* and *BRAF* mutation status was evaluated using qPCR.

**Results:**

We detected activating mutations in *NRAS* gene (codons: 12 and 61) in 7 out of the 18 (38.9%) patients. No *BRAF* mutations were found.

**Conclusion:**

Our study, the first molecular analysis of large/giant CMN in Polish patients, supports the hypothesis that *NRAS* mutation in codon 61 are frequent, recurrent mutations in large/giant CMN. Moreover, we show, for the first time, that *NRAS* mutations in codon 12 (p.Gly12Asp) can be also detected in giant CMN. The exact role of these genetic alterations in CMN formation remains to be elucidated.

## Introduction

**Figure 1:**
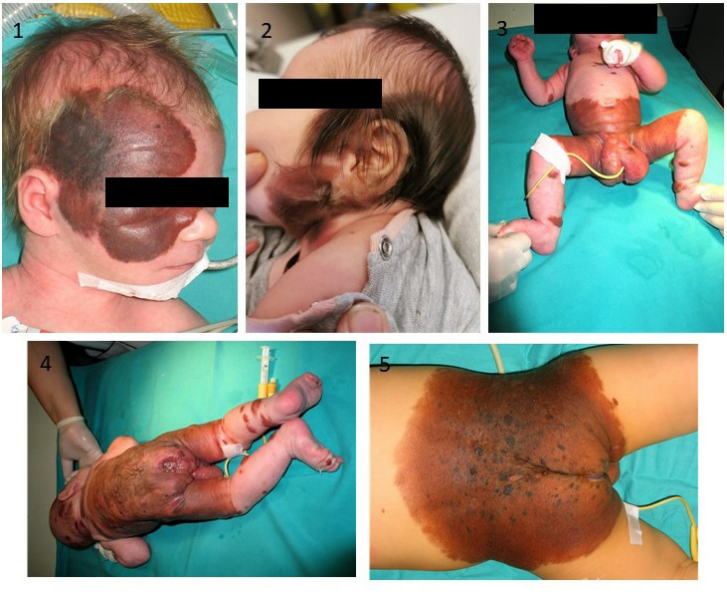
Examples of CMN: head localisation of the nevi (1, 2), anterior and posteriori view of neonate with bathing trunk localisation of the nevus before first operation (3, 4), neonate with back localisation of main nevus (5). *Photographed by* Marcin R. Szulżycki.

Congenital melanocytic nevi (CMN) are defined as neural crests derived benign naevomelanocytic proliferations being present at birth or emerging in the first weeks of life (Fig. 1). Uniquely, CMN can be present beyond the skin surface, spreading to the deep dermis, subcutaneous fat, fascia, or muscle, and the histological characteristics may be heterogeneous within single nevus [1]. The pigmentation of nevi can range from tan to dark brown, and clinically CMNs may vary considerably with respect to size, morphology, texture, and location [2]. CMNs can be distinguished by size: small, medium, large, and giant. Although initially the actual size of CMN was commonly evaluated at its current size, projected adult size (PAS) is now mostly used. This term refers to the largest diameter reached in adulthood and is calculated by multiplying actual CMN size by factors 1.7, 3.3, and 2.8, depending on lesion localisation (head, legs, and either trunk or arms and feet, respectively). According to this definition, large CMN (LCMN) have PAS ≥ 20 cm, while giant CMN (GCMN) are assigned if PAS exceeds 40–50 cm [3]. Some researchers also classify nevi covering over 2% of body surface area as LCMN or GCMN and nevi that cover a large portion of a major anatomical site as LCMN [2]. Large and giant CMN usually localise on the trunk, proximal parts of the limbs, scalp, or neck. GCMN often have “bathing trunks” and “glove stocking” distributions and are accompanied by multiple smaller satellite lesions, recently being called accompanying CMN [4]. The incidence of LCMN and GCMN is low (1/20,000 and 1/500,000 births, respectively); they are, however, considered a major health problem as they are connected with much higher risk of melanoma, neurocutaneous melanosis, other malformations of central nervous system, and greater therapeutic challenges [3]. For example, the lifetime risk of having melanoma for each individual with CMN equals 1–2%, while for those with larger CMN, the risk is higher and reaches 5–15% [4, 5]. Although the size of CMN was regarded as the single risk factor of those disorders for a long time, in 2013 an interdisciplinary group of experts elaborated a novel classification system that comprises more parameters to characterise CMN and determine risk of adverse events [6]. This classification includes also several satellite nevi in the first year of life (or actual), anatomic localisation, colour heterogeneity, surface rugosity, hypertrichosis, and presence of dermal or subcutaneous nodules. Nevertheless, as the authors themselves admit, the classification system would benefit greatly if genetic characteristics were part of it.

In recent years, few gain-of-function somatic variants have been reported as responsible for prenatal proliferation of melanoblasts. In particular, variants of codon 61 in *NRAS* were repeatedly detected in large and giant nevi, while *BRAF* p.Val600Glu variant in small to medium CMNs was reported in several studies [7]. Of note, the same variants as those described in CMNs are known molecular lesions detected in melanomas. However, unlike melanomas, in the case of CMNs, they seem to be single genetic abnormalities leading to minor gene dysregulations only [8]. Hence, further research on CMNs could shed light on melanoma investigation and also could reinforce novel therapeutic strategies of these disorders. The aim of the present study was to analyse the variant status of *NRAS* and *BRAF* genes in large and giant CMN resection specimens in a group of Polish patients.

## Material and methods

The archived affected skin samples taken from 18 children with large or giant congenital melanocytic nevi treated in Clinic of Surgery of Children and Adolescents Institute of Mother and Child in Warsaw in the years 2006 to 2017 were qualified for inclusion in the study. Localisation of main nevus was bathing trunk (five cases), back (seven patients), and head and/or neck in six patients. Age during first surgical intervention ranged from 1 month to 15 years. Surgical treatment was completed in 11 children (number of surgical procedures ranged from 2 to 21). Histological examination of excised nevus revealed compound nevus in all patients, but in two cases, in consecutive evaluations, melanoma arising in the nevus was additionally diagnosed. Accompanying neurocutaneous melanosis (NCM) was confirmed in six children (Table 1). Comprehensive clinical characteristics of the majority of our patients were published previously [9,10]. Resection specimens were taken from major lesions during surgery and fixed in formaline following paraffin embedment (FFPE samples). The samples were evaluated by a pathologist who marked regions containing the highest load of melanocytes, which were macro-dissected prior to DNA isolation. The material was collected and fixed in the years 2006 to 2017, therefore the majority of them were archive samples.

**Table 1:** Characteristics of patients and results of molecular investigation

Patient No	Year of birth/sex	Localization of nevus	Melanoma arising in the nevus	Neurocutaneous melanosis (NCM)	Age during first operation	Number of operations	Treatment completed	CMN size	Satellite nevi	*BRAF variants detected*	*NRAS variants detected*
**1**	2011/m	B			6/12	5	+	>20 cm	+	none	p.Gln61Lys
**2**	2012/m	H		+	7/12	4		> 0,5% PAS	0	none	none
**3**	2012/m	H			1/12	2	+	0,5 % PAS	0	none	p.Gln61Lys
**4**	2009/f	BT			4y	6		> 20 cm	++	none	none
**5**	1989/f	N			15y	4	+	> 20 cm	0	none	none
**6**	2003/f	B			1/12	5	+	> 20 cm	+	none	none
**7**	2007/f	BT		+	1/12	14		> 20 cm	0	none	none
**8**	2011/m	B			3/12	2	+	> 20 cm	0	none	p.Gln61Lys
**9**	2009/f	B			4/12	8	+	> 20 cm	+	none	none
**10**	2007/f	H			2y	8		> 0,5% PAS	+	none	p.Gln61Lys
**11**	2011/f	B			3/12	5	+	> 20 cm	+	none	p.Gly12Asp
**12**	2006/m	BT		+	4/12	21		> 20 cm	nd	none	none
**13**	2009/m	B		+	2y	5		> 20 cm	++	none	p.Gln61Lys
**14**	2002/m	BT			2y	10	+	not assessed	0	none	p.Gln61Arg
**15**	2012/m	HN			3/12	2		> 0,5% PAS	0	none	none
**16**	2010/f	B		+	6/12	4	+	> 20 cm	+	none	none
**17**	2006/f	BT	+	+	6/12	13	+	> 20 cm	nd	none	none
**18**	2008/m	H	+	+	1/12	3	+	> 0,5% PAS	-	none	none

m = male f = female BT = bathing trunk B = back H = head N = neck ND = no data PAS = projected adult size none: refers to spectrum of variants analyzed within the frames of this study

DNA was extracted with the Qiagen QIAamp® DNA Mini-Kit, according to manufacturer instructions. Variant status in *NRAS* and *BRAF* was analyzed using qPCR (EntroGen, *NRAS* Variant Analysis Kit and *BRAF* Variant Analysis Kit II for Real-Time PCR). The following variants were screened for: *NRAS*: p.Gly12Cys (c.34G>T), p.Gly12Ser (c.34G>A), p.Gly12Ala (c.35G>C), p.Gly12Asp (c.35G>A), p.Gly12Val (c.35G>T), p.Gln61Lys (c.181C>A), p.Gln61Arg (c.182A>G), *BRAF*: p.Val600Glu (c.1799T>A), p.Val600Lys (c.1798_1799GT>AA), p.Val600Asp (c.1799_1800TG>AT), p.Val600Arg (c.1798_1799GT>AG), p.Val600Met (c.1798G>A), p.Val600Gly (c.1799T>G). The names are given according to RefSeq: *NRAS*: NM_002524.5 (NP_002515.1) and *BRAF*: NM_004333.6 (NP_004324.2).

## Results

Clinical and molecular characteristics of the patients are depicted in Table 1. Overall, we detected variants in 7/18 (38.9%) of the patients. All of them were found in *NRAS* gene, predominantly in codon 61 i.e. p.Gln61Lys and p.Gln61Arg in 5 and 1 cases, respectively. In one case, p.Gly12Asp was identified. In none of the patients were *BRAF* variants identified. In 1/6 of patients with neurocutaneous melanosis (NCM) *NRAS* p.Gln61Lys variant was detected, whereas no variants were identified in the two cases diagnosed with melanoma (Table 1).

## Discussion

This study represents the first molecular study of large and giant CMN performed in Polish patients. In this study, we focused on detection of variants in the two genes: *NRAS* and *BRAF*. The *NRAS* and *BRAF* encode the GTPase NRas protein and serine/threonine-protein kinase B-raf, respectively, which are among the key proteins of the RAS/RAF/MEK/ERK signal transduction pathway involved in controlling cell growth and behaviour. Variants in genes encoding proteins of RAS/ RAF/MEK/ERK pathway are well known factors involved in melanomagenesis and its progression. Moreover, the pathogenic variants in *NRAS* and *BRAF* were also identified in the other melanocytic neoplasms, including congenital melanocytic nevi [11]. In fact, the frequency of variants in these genes is not equal, and in the case of larger CMN, *NRAS* variants occur more often, while *BRAF* variants are found mostly in small (<1.5 cm) and medium (1.5 cm–19.9 cm or <20 cm PAS) CMNs [7,12,13]. Moreover, earlier observations by S. Polubothu et al. indicating that *NRAS* and *BRAF* hotspot activating variants are almost mutually exclusive in malignant tumours, including melanoma, seems to be true also in CMN. Furthermore, the same study showed that *NRAS* and *BRAF* variants correlate with distinct nevi phenotype and histological findings. The authors state that their observations reflect the higher tolerance to *NRAS* variant early in embryogenesis and the fact that *NRAS* has several effector pathways, only one connected with *BRAF*, which may contribute to the observed phenotype [7]. Last but not least, considering the fact that mosaic *NRAS* and *BRAF* pathogenic variants are abundant in CMN, the researchers generally agree that they initiate congenital nevus development [14], but they do not trigger malignancy on their own [15]. Clearly, the molecular aetiology of CMNs needs further investigation.

In our group, *NRAS* p.Gln61Lys was the most prevalent variant, which is concordant with data reported so far [8]. The codon 61, where a catalytic residue required for efficient GTP hydrolysis locates [16], is a known variant hot spot, where variants are found most frequently both in melanoma and in the CMN [7,17]. Moreover, p.Gln61Lys tend to be the main variant in CMNs, though in melanoma the observed codon 61 variants distribution is more diverse [17].

Overall, we detected *NRAS* variants in 38.9% (7/18) of the patients, which is lower compared to other studies where the overall variant detection rate in giant/large CMN ranged from 70-95% [5,7,8,13,18]. This may be due to the fact that DNA was isolated from old, achieved paraffin-embedded samples that could have had an impact on the DNA integrity. The genome was not amplifiable with respect to NGS approach (data not shown), which highly limited the extent of our analyses. On the other hand, novel data emerged showing that point variants in other genes and fusion transcripts may also be involved in large/giant CMN aetiology [8,19]. Dessars et al. detected *ZEB2-ALK* and *SOX5-RAF1* gene fusions in two cases of giant CMN and point mutations in *KRAS* and *PIK3CA*. The somatic variant in *PIK3CA* and *GGNBP2-MYO19* gene fusion were also found in cases with *NRAS* variant [8].

Hence, we cannot exclude that at least some of our patients harbour other variants as well. In one patient, we found the *NRAS* p.Gly12Asp variant, which similarly to substitutions of Gln61, is a well-known oncogenic activating variant, but, to the best of our knowledge, it was never reported in CMN so far. Interestingly, it was shown that in mouse models, induction of *NRAS* p.Gly12Asp expression in embryonic melanocytes cause congenital melanocytic lesions, in one case being described as reminiscent of human blue nevi [20,21]. Importantly, both groups pointed out that although somatic *NRAS* p.Gly12Asp variant can induce CNS’s melanoma in mice, it does not seem to have any or very little potency to induce cutaneous melanomagenesis in mice. In fact, in one study melanoma was detected in one mouse from 29 models with *Nras* p.Gly12Asp variant in comparison to 14/20 mice with *Nras* p.Gln61Lys variant [21]. Interestingly, it was also shown in *in vitro* studies that when a conditional knock-in allele with *Nras* p.Gly12Asp mutation was expressed in hematopoietic lineage, it efficiently induced a myeloproliferative syndrome [21]. Moreover, according to AACR GENIE, in human the *NRAS* p.Gly12Asp was detected in acute myeloid leukaemia, colon adenocarcinoma, colorectal adenocarcinoma, rectal adenocarcinoma, acute myeloid leukaemia with myelodysplasia-related changes and in melanoma patients [22]. *NRAS* p.Gly12Asp is clearly associated with malignancy; however, further research is needed to identify the other molecular factors that are co-involved. Here, we show for the first time that *NRAS* p.Gly12Asp can be detected in patients with giant CMN. At the age of 8, the patient harbouring this variant did not show any symptoms from the central nervous system. Although the biological impact of variant localisation within *NRAS* seems plausible [21], we did not observe any significant differences between our patients with respect to major clinical findings (i.e. localisation of nevi, presence of satellite lesions, melanoma, and neuromelanosis) and molecular results.

In summary, our results indicate that *NRAS* variants in codon 61 predominate in our children group with large/giant CMN, which is in line with previous findings, and further supports the hypothesis that this is a recurrent somatic variant in this lesion type [13]. In the future, molecular research evaluating other genes (e.g. by NGS approach) with altered expression in CMN could provide more insights into molecular pathogenesis of giant CMN.

### Key points

1. We present the results of the first mutational analysis of large/giant congenital melanocytic nevi in Polish patients.
2. Our results indicate that *NRAS* mutations in codon 61 predominate in a group of Polish children with large/giant congenital melanocytic nevi.
3. Although further research is needed, considering worldwide trends toward precise medicine, our study is in line with public health policy. Within the next few years molecular studies may be included in a standard diagnostic/monitoring procedure of large/giant congenital melanocytic nevi.

### Compliance with ethical standards

All procedures performed in studies involving human participants were in accordance with the ethical standards of the institutional and/or national research committee and with the 1964 Helsinki declaration and its later amendments or comparable ethical standards.

Informed consent was obtained from all individual participants included in the study.
